# Effect of lingual-based flap design on postoperative pain of impacted mandibular third molar surgery: Split-mouth randomized clinical trial

**DOI:** 10.4317/medoral.23666

**Published:** 2020-07-19

**Authors:** Brevan Hassan, Nuraldeen Maher Al-Khanati, Haytham Bahhah

**Affiliations:** 1 Department of Oral and Maxillofacial Surgery, Faculty of Dental Medicine, Damascus University, Damascus, Syria; 2 Department of Oral and Maxillofacial Surgery, Faculty of Dentistry, Syrian Private University, Damascus, Syria

## Abstract

**Background:**

The extraction of third molars is one of the most common procedures in oral and maxillofacial surgery clinic. Surgical extraction involves the manipulation of both soft and hard tissues, so the patient usually experiences pain, swelling, and trismus in the immediate post-operative period. Several studies have been conducted using different types of surgical flaps to provide access with the least possible damage of soft tissues. Designing and implementing an optimum flap, which provides easier technique, better visibility, minimal post-operative complications, and best healing, is an aspired goal of every oral surgeon. This study aimed to compare lingual-based four-cornered flap with conventional triangular flap, and to evaluate its effect on post-operative pain after surgical extraction of impacted lower third molars.

**Material and Methods:**

Seventeen patients (age ranged from 19 to 26 years) with bilateral, symmetrical impacted lower third molars (n=34) were included in the study. This was a randomized clinical trial with a split-mouth design. The impacted molars were assigned randomly, by coin flipping, to two groups: Case side with lingual-based four-cornered flap (Group A), and control side with conventional triangular flap (Group B). Away from the incision, the prognosis, surgical intervention, and postoperative procedures were exactly the same for the two groups. Postsurgical pain was assessed for 5 days using visual analogue scale (VAS) and by recording patients need for analgesics on a daily basis. Patients were also evaluated via a self-reporting questionnaire, i.e. Postoperative Symptoms Severity (PoSSe) scale, administered on the seventh postoperative day.

**Results:**

Pain scores recorded in Group A were found to be significantly lower as compared to pain scores in Group B in the 5 postoperative days (*P*<0.05). Total analgesic intake in Group B was significantly higher (*P*<0.05). PoSSe scores were lower in Group A, however, this difference was insignificant (*P*>0.05).

**Conclusions:**

According to the data of the current study and within its limits, it appeared that lingual-based four-cornered flap design was superior to the conventional triangular flap regarding the postsurgical discomfort and pain.

** Key words:**Impaction, third molar surgery, flap design, pain, PoSSe scale, split-mouth, RCT.

## Introduction

The extraction of impacted third molars is one of the most common procedures in dental clinic. This is because of the relative high prevalence of impaction. About 90% of people have third molars (1). More than 57% of patients have at least one impacted third molar (2). This high prevalence of impaction is associated with genetic and environmental factors (2,3).

Indications of third molars extraction include caries (in partially-erupted third molars and/or in their adjacent second molars), periapical pathology, recurrent pericoronitis, infection (i.e. abscess or osteomyelitis), internal and/or external root resorption (of third molar or adjacent tooth), fracture of mandibular angle, trauma and fracture of tooth, extraction for dental autotransplantation, orthodontic reasons, and periodontal disease (4-6). Although great controversy exists about prophylactic removal of third molars, there is now considerable evidence supporting the extraction of symptom-free impacted molars (4,7,8).

Surgical extraction of mandibular third molars involves mucoperiosteal flap reflection with or without bone removal. The postoperative complications that may occur following the extraction of impacted third molar include pain, edema, trismus, decreased masticatory function, dry socket and neurological complications (9,10). Pain and swelling are triggered by an inflammatory response in the surgical area leading to vasodilation and arrival of strong pro-inflammatory mediators (11). Severity of these complications may differ in different patients and not necessarily be presented in all patients (11).

Many researchers used different types of surgical flaps trying to provide access with the least possible soft tissue damage (12-14). Incisions should be designed to provide good blood supply, good access to allow adequate vision and space for instrumentation, to protect the soft tissues, minimize trauma and permit repositioning and reattachment of the flap. It must be a full-thickness incision. The outcome of surgery is affected by various factors such as flap design, surgeon’s experience, instrumentation, amount of bone removal, difficulty of extraction, sectioning of crown and/or roots, suturing techniques, patients age, host response, race and gender (15). One must unify all other variables to evaluate the effect of certain flap design on surgical consequences.

This study aimed to compare lingual-based four-cornered flap with triangular flap of modified Ward’s incision in regard to the post-operative pain of impacted lower third molar surgical extractions.

- Abbreviations

VAS= Visual Analogue Scale, PoSSe=Postoperative Symptoms Severity, min= minutes, rpm= round per minute, RCT= Randomized Clinical Trial.

## Material and Methods

The patients were enrolled in this split-mouth randomized clinical controlled trial and treated according to the prospective study protocol. The study protocol was reviewed and approved by Research Ethics Committee of Damascus University (Registration No. 2083). Seventeen patients, who attended oral and maxillofacial surgery department in Damascus University for surgical extractions in the period from December 2018 to May 2019, were included in this study. All patients provided informed consent. Inclusion criteria were as follows: 1) healthy patients with asymptomatic, bilateral, symmetrical impacted mandibular third molars; 2) impacted molars must be mesioangular and easy to extract, i.e. not too deep, too close to second molar, close to mandibular canal, nor having ankylosed, widely divergent, or bulbous roots; 3) good oral hygiene; 4) age range between 19 and 26 years; 5) absence of any medical condition that may contraindicate surgery, such as uncontrolled or poorly-treated diabetes, history of radiotherapy and/or chemotherapy, blood disorders, and immunosuppression.

**Figure 1:**
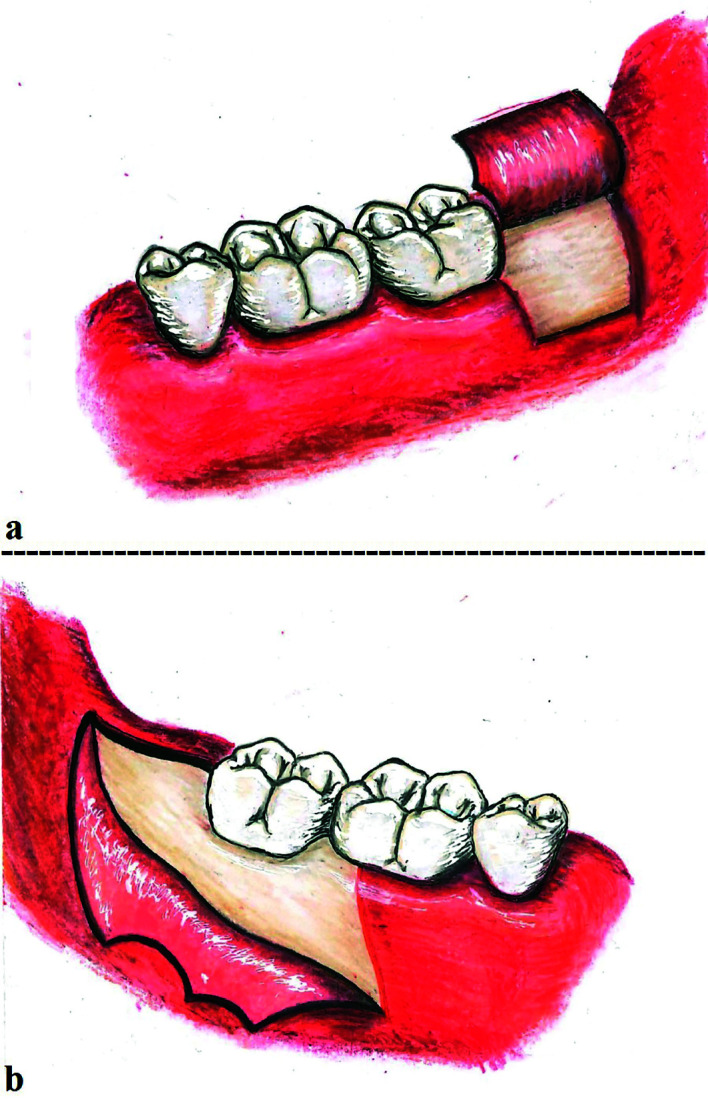
An illustration showing the lingual-based four-cornered flap used in Group A- case side (a) and the triangular flap used in Group B- control side (b).

Preoperative evaluations of surgical potential complexity were done with aid of Pernambuco index (16). Impacted molars had to score 12 points as a maximum according to this scale to classify their surgical difficulty as “low” (16). Any case with a score of more than 12 had to be excluded. The inclusion rate was 100%. Each impacted molar was randomly assigned into one of the two study groups using coin flipping by third party, i.e. either into Group A (case side) or Group B (control side). Away from the incision, the prognosis, difficulty index and angulations of third molars, surgical interventions, and postoperative procedures and medications were exactly the same for the two groups. The only single difference was in surgical flap designing. In the case side lingual-based four-cornered flap was used (Fig. 1), while conventional triangular flap design was implemented in the control side (Fig. 1).

**Figure 2:**
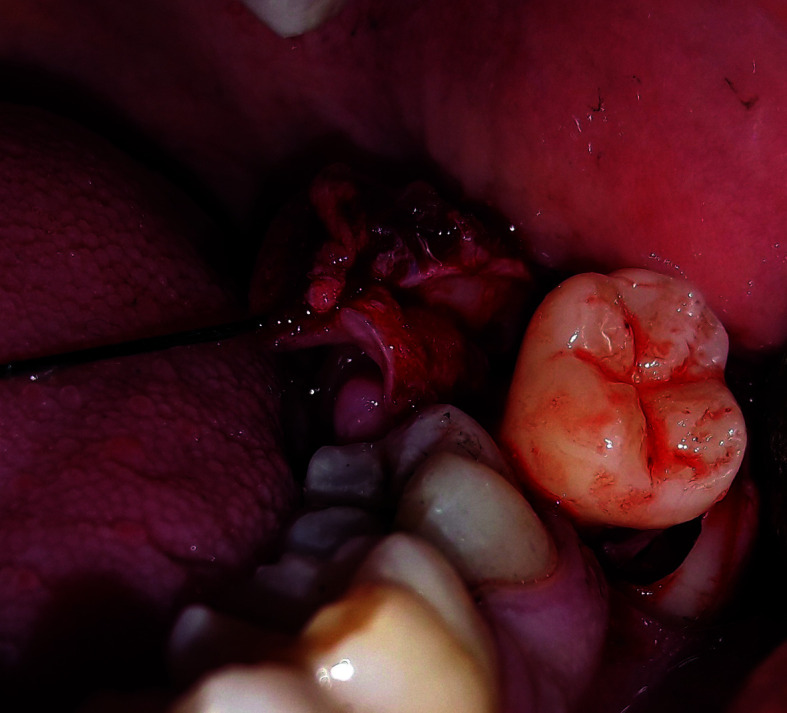
Elevated full-thickness lingual-based four-cornered flap retracted with silk suture to the lingual side (at the moment the impacted lower third molar was extracted)- Group A.

The lingual-based four-cornered flap was designed and marked on the mucosa using special pencil so that the flap base was drawn from the mid-point of distal surface of lower second molar to a point 1.5-2.5 cm posteriorly. A vertical incision was started from this posterior point 2-2.5 cm buccally toward the depth of buccal vestibule. Then, the incision is extended anteriorly 1.5-2 cm parallel to the flap base. Another vertical incision toward the disto-buccal angle of the second molar crown is made, and extended with a sulcular incision to the distal surface mid-point. Both flap base and apex had two corners each. This made the flap a four-cornered flap. A full-thickness mucoperiosteal flap was reflected afterward to expose the impacted lower third molar. 4-0 silk suture was used to retract the flap from the lingual side to ensure best possible access and field of view (Fig. 2).

The triangular flap, used in the control side, began with anterior vertical incision from the disto-buccal corner of the lower first molar crown to the bottom of buccal vestibule alongside the mesio-buccal cusp of that tooth. A horizontal sulcular incision was made and extended posteriorly to the external oblique ridge (Fig. 1).

All surgeries were done by the same surgeon and surgical team under local anesthesia of 2% lidocaine with epinephrine (1:80,000). Requisite bone removal was performed using carbide round bur on low-speed straight handpiece (20,000 rpm) with copious cooling by saline. Flip of a coin in order to achieve simple randomization was utilized twice; once to distribute patient’s impacted molars on both sides into two groups, and one more time to choose which side to begin with. A convalescent period of at least 20 days between the two surgical extractions (right and left sides) for each patient was given to ensure that symptoms of the first surgery totally disappeared, and to minimize effect of pain memory. With the aid of timekeepers, two durations were recorded in minutes; the total surgery duration from the beginning of incision to the last stitch sutured; and time particularly taken for bone removal.

After surgery, all patients received fixed instructions regarding local homeostasis, cleansing, food and medical prescription. Postoperative medication included painkiller and mouthwash, and did not include any antibiotic. Patients were asked to rinse with 0.12% chlorhexidine solution twice daily for 10 days, starting the next day after surgery. The only allowed analgesic was oral Tablets of paracetamol 500 mg. Patients were instructed to take one Tablet as necessary, with a maximum of 6 Tablets a day. It was not allowed to add any other analgesic or medication to the prescription. Taking into account the ease of extraction predetermined in the inclusion criteria and the followed aseptic surgical procedures, there was no need for antibiotics. In addition, antibiotics and anti-inflamatory drugs could have been a variable, thus might have affected the results. So only a drug with less potent anti-inflammatory actions, i.e. paracetamol, was chosen for fair assessment. Patient analgesic need was assessed by asking the patient to write down the number of painkiller Tablets taken each day postoperatively for 5 days. A segmented 11-item numeric version of visual analogue scale (VAS) was used in this study. Patients were requested to select a number (0-10 integers) that best reflected the intensity of their pain on a horizontal bar. The scale was combined with descriptive 6-levels segments; 0= no pain, 1-3= mild pain, 4-5= moderate pain, 6-7= painful, 8-9= very painful, 10= unbearable pain. Patients were asked to report pain intensity at the worst time of the day, for 5 days post-surgery. Clinical follow-up and removal of sutures were done after one week. On that day, a comprehensive Postoperative Symptoms Severity (PoSSe) questionnaire was given to each participant. It consisted of questions that are commonly used in assessment of patients who have had third molar surgeries. These questions are related to seven subgroups that include patient’s capability to enjoy food, speak properly, sensation, appearance, pain, sickness, and interference with everyday activities (17).

G*Power software V3.1 (Univesität Kiel, Germany) was used to perform calculations regarding sample size. It is noteworthy that split-mouth design requires smaller sample size than other RCT designs (18). The effect size and required sample size was calculated using the discrepancy in means of total analgesics’ consumption for 5 postoperative days in Group A (2.428 ±0.786) and in Group B (3.428 ±0.975) obtained from pilot study which was conducted and included 14 surgical extractions (n=14) in a total of 7 patients fulfilling the same eligibity criteria. An periori power analysis in G*Power V3.1 recommended a minimum sample size of 28 surgeries (14 patients), when assuming 80% power and α of 0.05. Statistical analyses were performed using Statistical Package for the Social Sciences for Windows V19 (SPSS Inc, Chicago, IL, USA). Statistics included descriptive and comparative tests of variables. The Mann-Whitney U and independent t-student tests were mainly applied to evaluate differences in the values between the two study groups. *P* values of less than 0.05 were considered significant.

## Results

**Figure 3:**
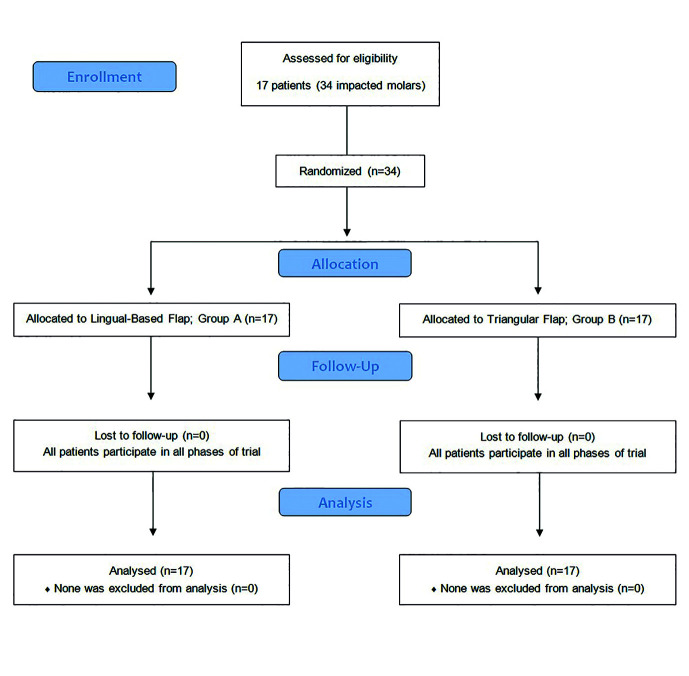
Flow diagram of the progress through stages of this split-mouth randomized trial.

A total of 34 impacted mandibular third molars (n=34) in 17 patients (7 males and 10 females) were included in the study and subsequently extracted. The progress of subjects in phases of this RCT is shown (Fig. 3). The mean patient age was 21.4 years (± 1.8). Variables distribution among study groups is shown (Table 1). No significant difference appeared between the two groups regarding predicted surgical difficulty (*P*=0.233). Neither crown nor roots sectioning was necessary in any of cases. No complications were associated with the 34 surgeries, other than that few stitches were observed to be torn only in Group B. Duration of surgical extraction in Group B was significantly shorter than in Group A (*P*<0.001). However, the mean bone removal duration in Group B was slightly longer with no significant difference between the two groups (*P*=0.748).

All 34 surgeries were followed up and included in the statistical analysis with no missing data (Fig. 3). There were significant statistical differences in pain intensity (VAS scores) between the two groups in all 5 postsurgical days; VAS scores in Group B were significantly higher (*P*<0.05; Table 1). The number of analgesic Tablets taken by patients each day after surgery for 5 days was significantly lesser in Group A in comparison with analgesic intake in Group B (*P*<0.05; Table 1). Participants’ scores on the full PoSSe scale showed no significant differences between Group A and Group B (*P*>0.05; Table 1). Detailed scores on each question of the scale are comparable and arranged in Table 2. Only the last subscale question concerning “how badly pain affected patient’s life” showed a statistically significant difference between the two groups (*P*=0.008; Table 2).

**Table 1:**
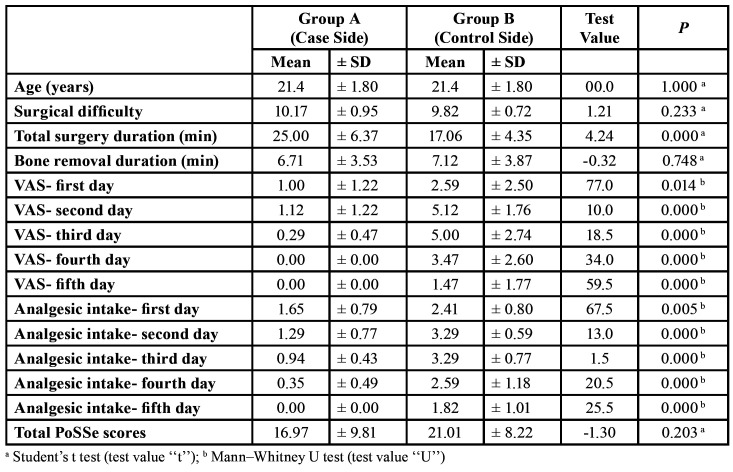
Distribution of variables and statistical tests results and comparisons; Patients age, predicted surgical difficulty, surgery time, visual analogue scale (VAS), analgesic intake, PoSSe scores.

**Table 2:**
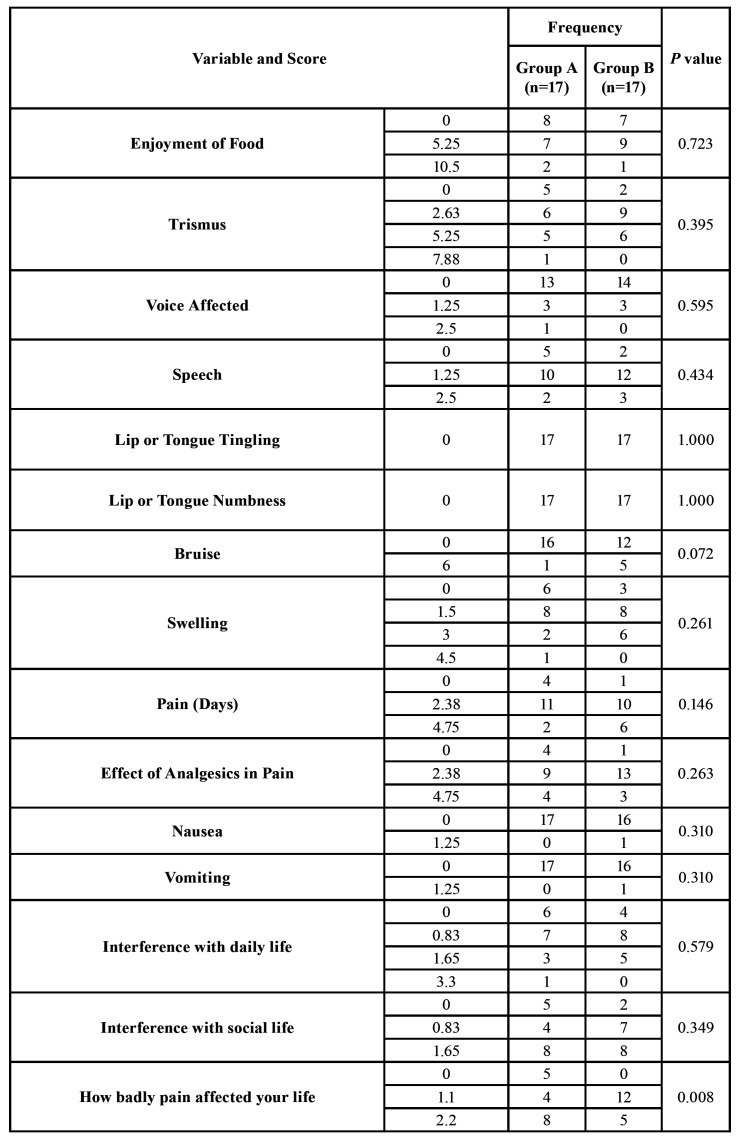
Comparison of scores of Postoperative Symptoms Severity (PoSSe) scale between both groups of the current study showing subscale questions differences.

## Discussion

Surgical extraction of impacted lower third molar is one of the most frequent procedures in oral and maxillofacial surgery. It demands sound understanding of surgical principles to be performed with less trauma and postoperative pain as possible (19). Different surgical techniques have been introduced to perform atraumatic procedures and to reduce postoperative pain (19,20).

The lingual nerve pathway in the retromolar region of the mandible is often close to the traditional surgical incision used for surgical extraction of impacted lower third molars (21). This increases the risk of neurological complications after impaction surgeries; i.e. lingual nerve injury and subsequent continuous numbness in the tongue. The obvious principal benefit of using a lingual-based flap, when extracting a lower impacted third molar, is to ensure the best vision and eliminate the possibility of lingual nerve damage (22). This is logical and confirmed by clinical trials (13,22). The present study aimed to investigate the effect of this type of flaps on another important variable, one of the most annoying consequences of mandibular third molar surgery; i.e. postoperative pain.

Two different surgical flap designs were compared; lingual-based four-cornered flap and triangular flap via modified Ward’s incision. Both flap designs were always carried out within the mouth of each participant after randomly allocating them to patient’s right and left sides. This split-mouth study design removes so much of inter-subject variability, such as gender, race, age, host response, and pain tolerance, and so improves study power (15). Although there was a potential effect of chlorhexidine oral rinsing on pain and other surgical outcomes, it was preferred to be prescribed for ethical reasons to maintain a good level of oral health postoperatively. In any case, adherence to one single exact medical prescription and fixed postsurgical instructions given to all patients equally in addition to the presence of one and the same surgeon and surgical assistants in all cases, all of these would help in controlling effect of different variables. Moreover, authors included only asymptomatic cases to ensure the state of zero pain level pre-surgery and eliminate an additional confounding factor. The majority of these symptom-free impacted molars were referred from orthodontic department for extraction. Orthodontic reasons for impacted mandibular third molar extraction include: preparation for orthognathic surgery, prevention of late incisor crowding, and before molar distalization.

Post-operative pain of impacted lower third molar surgery is developed due to localized inflammation in the surgical area. Surgical extraction, conjugated tissue injury and cellular destruction provoke releasing and production of several biochemical mediators, e.g. histamine, bradykinin and prostaglandins, which are involved in pain process (14). Although it is not that simple, we can assume based on the above: the smaller the injury, the lesser the pain. The area of surgical intervention on soft tissues with lingual-based flap design is smaller than it with conventional triangular flap. Moreover, retracting the reflected flap toward its lingual base by silk suture is seemingly gentler and causing less damage to tissues than the conventional buccal retracting by Farabeuf retractor used with the traditional flap design. Farabeuf retractor seems to apply more force on the flap when compared to a silk suture. This may explain the results of the present study that showed significant lower pain intensity levels and significant lower patients’ analgesic need in the group of lingual-based flap during the postoperative follow-up period (*P*<0.05).

These findings are in accordance with the outcome of Rai *et al* (22). Although the used flap was somewhat different from the one used in this study, they found that lingual-based four-cornered flap is better than the conventional triangular flap regarding pain, swelling and dry socket (22). Nageshwar found similar results (13). After comparing conventional modified envelop incision and comma-shaped incision designs in 100 patients undergoing impaction surgeries, he found that patients with the smaller flap experienced less pain (13). In contrast, Yolcu and Acar’s results showed initial greater pain in patients treated with lingually based triangular flaps when compared to patients treated with buccally based triangular flap (23). As is obvious in the same previous mentioned study, size of the used lingual-based flap is larger than the comparable one (23). Also, it is clearly different from the flap used in Group A of the current study.

The total surgery duration was significantly elongated when the lingual-based flap is considered (*P*<0.001). This was due to more time needed for suturing in Group A than in Group B. The Closed sockets in Group B were often broken down after few days and healed by secondary intention. Conflicting opinions regarding the primary and secondary wound healing have been expressed in literature (24,25). Although partial closure of the flap reduces the operating time, healing by secondary intention is slower than by primary intention (26,27). In secondary healing, extraction socket remains in communication with saliva and oral fluids (24). Risk of alveolar osteitis development after extraction is higher in open healing (28). Wound healing, including pain, is worse when a high-tension wound is sutured (29). Disintegration of some stitches after a while in the control side, rather than the study side, indicates a greater tension on the wound edges in this group. This may participate in explaining the findings of this study.

Scores of full PoSSe scale showed that differences between the two study groups concerning the patient-reported severity of symptoms, e.g. trismus, swelling... etc, in total were insignificant (*P*=0.203). Since PoSSe scale is subjective, authors suggest that further research including objective parameters is needed to confirm that lingual-based four-cornered flap design has no effect on postoperative trismus and oedema. This result is in line with Glera-Suárez *et al*. who conducted a meta-analysis and found no clear differences in patient morbidity between different designs of surgical flap (30). However, PoSSe detailed scores showed significant difference in the answers of one question of the questionnaire that asked the patients to tell the degree the pain affected their lives between the two study groups (*P*=0.008).

## Conclusions

Although surgical procedures’ durations were significantly elongated with the implementation of lingual-based four-cornered flap, it seemed to be preferable over the modified Ward’s buccal-based triangular flap with regard to pain after surgical extraction of impacted mandibular third molars.

## References

[1] Rosa AL, Carneiro MG, Lavrador MA, Novaes AB Jr (2002). Influence of flap design on periodontal healing of second molars after extraction of impacted mandibular third molars. Oral Surg Oral Med Oral Pathol Oral Radiol Endod.

[2] Hashemipour MA, Tahmasbi-Arashlow M, Fahimi-Hanzaei F (2013). Incidence of impacted mandibular and maxillary third molars: a radiographic study in a Southeast Iran population. Med Oral Patol Oral Cir Bucal.

[3] Trakinienė G, Šidlauskas A, Trakinis T, Andriuškevičiūtė I, Šalomskienė L (2018). The Impact of Genetics and Environmental Factors on the Position of the Upper Third Molars. J Oral Maxillofac Surg.

[4] Hyam DM (2018). The contemporary management of third molars. Aust Dent J.

[5] Medina-Solís CE, Mendoza-Rodríguez M, Márquez-Rodríguez S, De la Rosa-Santillana R, Islas-Zarazua R, Navarrete-Hernández JD (2014). Reasons why erupted third molars are extracted in a public university in Mexico. West Indian Med J.

[6] Ahmed Asif J, Yusuf Noorani T, Khursheed Alam M (2017). Tooth Auto-transplantation: An Alternative Treatment. Bull Tokyo Dent Coll.

[7] McArdle LW, McDonald F, Jones J (2014). Distal cervical caries in the mandibular second molar: an indication for the prophylactic removal of third molar teeth? Update. Br J Oral Maxillofac Surg.

[8] Kalantar Motamedi MR, Heidarpour M, Siadat S, Kalantar Motamedi A, Bahreman AA (2015). Orthodontic Extraction of High-Risk Impacted Mandibular Third Molars in Close Proximity to the Mandibular Canal: A Systematic Review. J Oral Maxillofac Surg.

[9] Jerjes W, El-Maaytah M, Swinson B, Banu B, Upile T, D'Sa S (2006). Experience versus complication rate in third molar surgery. Head Face Med.

[10] Alqahtani NA, Khaleelahmed S, Desai F (2017). Evaluation of two flap designs on the mandibular second molar after third molar extractions. J Oral Maxillofac Pathol.

[11] Troiano G, Laino L, Cicciù M, Cervino G, Fiorillo L, D'amico C (2018). Comparison of Two Routes of Administration of Dexamethasone to Reduce the Postoperative Sequelae After Third Molar Surgery: A Systematic Review and Meta-Analysis. Open Dent J.

[12] Zhu J, Yuan X, Yan L, Li T, Guang M, Zhang Y (2020). Comparison of Postoperative Outcomes Between Envelope and Triangular Flaps After Mandibular Third Molar Surgery: A Systematic Review and Meta-Analysis. J Oral Maxillofac Surg.

[13] Nageshwar (2002). Comma incision for impacted mandibular third molars. J Oral Maxillofac Surg.

[14] Kumar B S, T S, M V, Raman U (2013). To compare standard incision and comma shaped incision and its influence on post-operative complications in surgical removal of impacted third molars. J Clin Diagn Res.

[15] Al-Khanati NM, Al-Moudallal Y (2019). Effect of Intrasocket Application of Manuka Honey on Postsurgical Pain of Impacted Mandibular Third Molars Surgery: Split-Mouth Randomized Controlled Trial. J Maxillofac Oral Surg.

[16] de Carvalho RWF, Vasconcelos BC (2018). Pernambuco index: predictability of the complexity of surgery for impacted lower third molars. Int J Oral Maxillofac Surg.

[17] Ruta DA, Bissias E, Ogston S, Ogden GR (2000). Assessing health outcomes after extraction of third molars: the postoperative symptom severity (PoSSe) scale. Br J Oral Maxillofac Surg.

[18] Pandis N (2012). Sample calculation for split-mouth designs. Am J Orthod Dentofacial Orthop.

[19] Sortino F, Cicciù M (2011). Strategies used to inhibit postoperative swelling following removal of impacted lower third molar. Dent Res J (Isfahan).

[20] Mobilio N, Vecchiatini R, Vasquez M, Calura G, Catapano S (2017). Effect of flap design and duration of surgery on acute postoperative symptoms and signs after extraction of lower third molars: A randomized prospective study. J Dent Res Dent Clin Dent Prospects.

[21] Behnia H, Kheradvar A, Shahrokhi M (2000). An anatomic study of the lingual nerve in the third molar region. J Oral Maxillofac Surg.

[22] Rai A, Rai M (2017). Lingual Based Four Cornered Flap for Third Molar Surgery. J Maxillofac Oral Surg.

[23] Yolcu Ü, Acar AH (2015). Comparison of a new flap design with the routinely used triangular flap design in third molar surgery. Int J Oral Maxillofac Surg.

[24] Maria A, Malik M, Virang P (2012). Comparison of primary and secondary closure of the surgical wound after removal of impacted mandibular third molars. J Maxillofac Oral Surg.

[25] Chaudhary M, Singh M, Singh S, Singh SP, Kaur G (2012). Primary and secondary closure technique following removal of impacted mandibular third molars: A comparative study. Natl J Maxillofac Surg.

[26] Gay-Escoda C, Gómez-Santos L, Sánchez-Torres A, Herráez-Vilas JM (2015). Effect of the suture technique on postoperative pain, swelling and trismus after removal of lower third molars: A randomized clinical trial. Med Oral Patol Oral Cir Bucal.

[27] Politis C, Schoenaers J, Jacobs R, Agbaje JO (2016). Wound Healing Problems in the Mouth. Front Physiol.

[28] Aydintug YS, Bayar GR, Gulses A, Misir AF, Ogretir O, Dogan N (2012). Clinical study on the closure of extraction wounds of partially soft tissue-impacted mandibular third molars. Quintessence Int.

[29] Song M, Zhang Z, Liu T, Liu S, Li G, Liu Z (2017). EASApprox® skin-stretching system: A secure and effective method to achieve wound closure. Exp Ther Med.

[30] Glera-Suárez P, Soto-Peñaloza D, Peñarrocha-Oltra D, Peñarrocha-Diago M (2020). Patient morbidity after impacted third molar extraction with different flap designs. A systematic review and meta-analysis. Med Oral Patol Oral Cir Bucal.

